# Factors associated with self-isolating and completing a contact follow-up program: a retrospective analysis of Ontario, Canada’s COVID-19 contact tracing initiative

**DOI:** 10.1186/s12889-025-24737-2

**Published:** 2025-11-26

**Authors:** Justin Thielman, Celina Degano, Samantha Gray, Andrea Chambers, Elaina MacIntyre

**Affiliations:** 1 Public Health Ontario, Toronto, ON Canada; 2 Dalla Lana School of Public Health, University of Toronto, Toronto, ON Canada

**Keywords:** Infectious disease, Contact tracing, Pandemic, Public health, Epidemiology, COVID-19

## Abstract

**Background:**

Contact tracing is an important tool in slowing the spread of infectious disease and preventing illness. People may face barriers to contact tracing adherence, depending on various personal and contextual characteristics. Thus, we examined a large-scale COVID-19 contact tracing initiative and compared adherence across several socio-demographic and exposure characteristics.

**Methods:**

We analyzed data for 130,255 participants in Ontario’s COVID-19 contact tracing initiative from September 2020 to December 2021. During contact follow-up calls, callers recorded whether contacts reported self-isolating and whether they completed follow-up. We performed unadjusted and adjusted logistic regressions to estimate the odds ratios (OR) and 95% confidence intervals (CI) of self-isolating and of completing follow-up, according to contacts’ age group, neighbourhood material resources, COVID-19 wave, exposure setting, region, and preferred language.

**Results:**

In the adjusted analyses, odds of completing follow-up decreased as neighbourhood-level material resources decreased, with OR = 0.57 (95% CI: 0.54–0.60) comparing highest resource areas to lowest. Compared to COVID-19 contacts living in the Greater Toronto Area, other Ontario regions had higher odds of completing follow-up, with ORs ranging from 1.19 (95% CI: 1.14–1.24) to OR = 1.91 (95% CI: 1.78–2.04). Contacts whose preferred languages were not English had lower odds of self-isolating (OR = 0.57, 95% CI: 0.38–0.84). The 0–4 and 5–11 year old age groups had lower odds of self-isolating than 20–29 year olds, with respective ORs of 0.60 (95% CI: 0.48–0.75) and 0.57 (95% CI: 0.48–0.67).

**Conclusions:**

In preparing for future pandemics, contact tracing programs may benefit from prioritizing additional supports for those who live in areas with fewer material resources, have language preferences other than English, and live in large metropolitan centres.

**Supplementary Information:**

The online version contains supplementary material available at 10.1186/s12889-025-24737-2.

## Background

Contact tracing is regularly used in infectious disease prevention and control to interrupt the chain of disease transmission [1]. Contact tracing involves identifying, notifying, and following up with close contacts of suspected or confirmed cases [2]. Large-scale contact tracing initiatives were widely used during the COVID-19 response, particularly before vaccines were widely available [3, 4]. as timely and thorough follow-up with close contacts of COVID-19 cases, with instructions to self-isolate and monitor for symptoms, can reduce COVID-19 transmission [3, 5]. 

For contact tracing to be effective, contacts must be informed of their exposure to a case promptly, before they become infectious [6]. Contacts must also adhere to requests to quarantine or self-isolate, monitor for symptoms and follow any additional public health guidance. In this paper, the term self-isolate will be used to describe COVID-19 contacts quarantining themselves. Jurisdictions often prioritized identifying high-risk contacts. Numerous factors may affect whether identified high-risk contacts can be reached by contact tracers and whether they adhere to instructions to self-isolate, including personal characteristics such as age, where the contact lives, and the timepoint in the pandemic [7, 8]. Both public health practitioners and researchers have recognized the need to better understand these challenges, to develop tailored resources and supports to facilitate individuals’ capability, opportunity and motivation to participate fully in the contact tracing process [8, 9]. 

Two indicators that can be used to monitor contact tracing outcomes include the proportion of contacts who comply with recommendations to self-isolate and the proportion of contacts who agree to follow-up and monitoring during the recommended isolation period [10]. However, few studies have been able to leverage administrative data sources from contact tracing programs to examine adherence to self-isolation and follow-up [7, 10, 11]. In the Canadian province of Ontario, Public Health Ontario developed a centralized Contact Tracing Initiative (CTI) to support Ontario’s 34 local public health units (PHUs) with COVID-19 contact follow-up within their regions [12]. Local PHUs were responsible for following up with cases, which involved identifying all close contacts for each case in their region. PHUs were also responsible for contact tracing, although they had the option to transfer any high-risk community contacts to the CTI for notification of possible COVID-19 exposure and/or follow-up for the remaining self-isolation period (up to 14 days). The CTI employed standardized call scripts, processes, and data collection, allowing for rapid scale-up of contact tracing and alignment with the province of Ontario’s guidance on contact management [13]. Consequently, the CTI was most useful for contacts who did not require specialized messaging. For example, healthcare workers, who often had different isolation rules to allow them to return to work, or people who were immunocompromised and had to isolate for longer, were not eligible for the CTI as they required more specialized messaging than the general public. However, school contacts were commonly submitted to the CTI, as they largely shared the same isolation requirements. Thirty-three of the 34 local PHUs used the CTI at some point during its operations, with participation rates varying across PHUs and over time. A detailed description of the CTI is provided in the supplementary appendix.

While unable to capture all contacts nor all aspects of a contact tracing program, the centralized database used by the callers provided an opportunity to analyze data to identify certain indicators of contact tracing effectiveness and examine the relationship between these indicators and contacts’ age, residential location, date of exposure, preferred language and exposure setting. We hypothesize that such an analysis can identify differences in indicators of contact tracing effectiveness related to these characteristics, which may identify how future contact tracing efforts can be tailored to meet the needs of diverse populations and various circumstances.

### Objective

Our primary objective was to examine whether sociodemographic and other contextual characteristics of COVID-19 contacts in the CTI were associated with two outcomes: contacts reporting adherence to self-isolation and contacts completing all required steps for follow-up in the CTI.

## Methods

### Study design

**Fig. 1:**
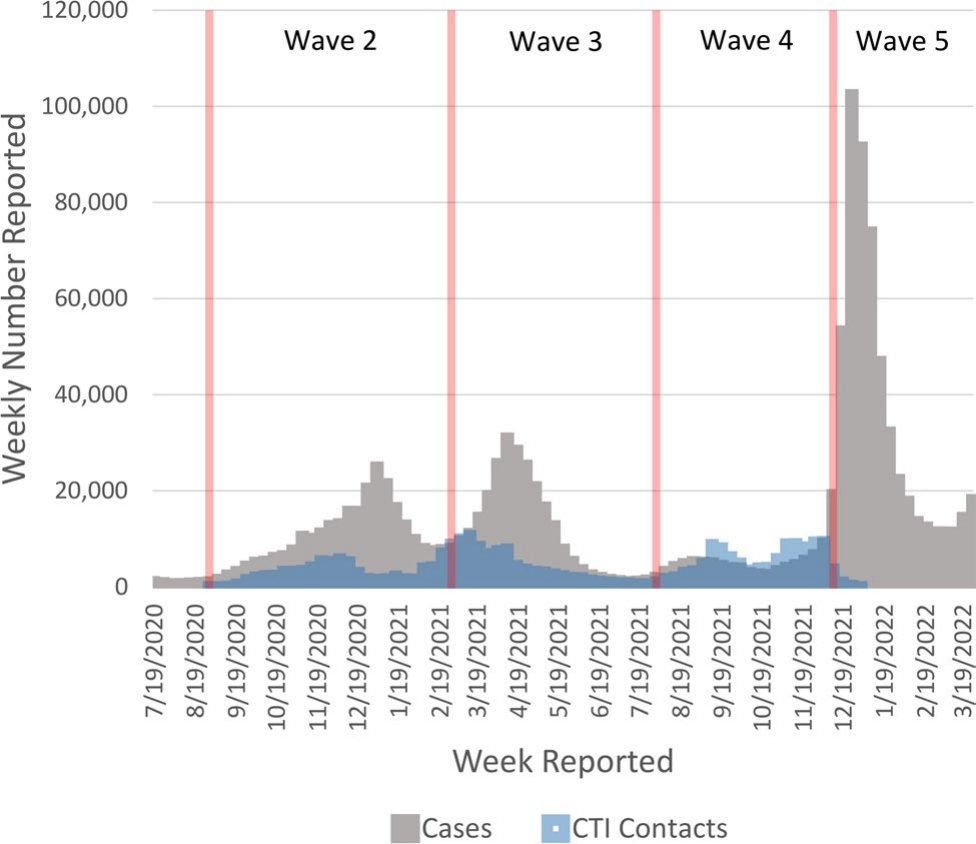
COVID-19 cases and contacts identified each week from July 2020 to March 2022, using Public Health Ontario’s Case and Contact Management system

This study is a retrospective analysis of COVID-19 contacts followed by Public Health Ontario’s CTI. The CTI transitioned to an electronic Case and Contact Management (CCM) system in September 2020 and operated until late February 2022, with a sharp decline in program use at the end of December 2021 due to the emergence of the Omicron variant in Ontario, which prompted Ontario’s contact tracing guidance to no longer require follow-up of most contacts, due to reprioritization of resources (Fig. 1). Therefore, the observation period for this study was September 1, 2020 to December 31, 2021. During this time, contacts in the program received multiple calls to monitor their symptoms during a self-isolation period of up to 14 days. This study was approved by Public Health Ontario’s Ethics Review Board and the Privacy Office.

Contact Tracing Initiative (CTI) totals were collected from a combination of Case & Contact Management (CCM) system data and pre-CCM data that were collected using Excel spreadsheets. Cases were identified from aggregate data from Public Health Ontario’s COVID-19 data tool, which tracked COVID-19 cases on a weekly basis and included options to download weekly case numbers for Ontario. Duplicate investigations were removed. The true number of contacts submitted by each PHU may be over or underestimated, due to system user error.

### Data sources

Study data came from the CCM system and was entered by Ontario’s local PHUs and by contact tracers during phone interviews. The only exception was the material resources quintiles, which were included by merging in a separate dataset: the Ontario Marginalization Index [14]. This is a data tool that includes four dimensions of marginalization: material resources, racialized and newcomer populations, age and labour force, and households and dwellings [14]. Our study used one of these dimensions: material resources. The material resources dimension is connected to poverty and refers to the inability of individuals and communities to access and attain basic material needs relating to food, clothing, and education [15]. Material resources quintiles were calculated at the census dissemination area level, with quintile 1 having the most resources and quintile 5 having the least. Dissemination areas are small geographic areas that typically contain 400–700 Ontario residents [16]. This index included values showing each area’s quintile of material resources. The vast majority (98.3%) of Ontario dissemination areas were assigned material resources quintiles in this dataset. Data were merged using a postal code conversion file that matches dissemination areas with postal codes [17]. Postal codes were entered as CTI data by local PHUs before submitting each contact, and were occasionally updated by callers during phone interviews. Postal codes were not mandatory information to collect during calls.

### Study eligibility

The study population comprised high-risk community contacts of COVID-19 cases who were identified by local PHUs and referred to the CTI for follow-up. The CTI was designed to provide support to PHUs, which often did not have capacity to follow up with the high number of COVID-19 contacts, especially during surges in the volume of contacts. The CTI was an optional program; local PHUs decided how many and which contacts they referred. Data on contacts who were not referred to the CTI were not captured, so we were unable to determine the proportion of contacts submitted to the CTI or factors that may have influenced submission. Therefore, the study population is not representative of a broader Ontario population. Additional details on the process of transferring contacts between PHUs has been published previously, including a flow diagram showing how contact follow-up and data collections flowed between PHUs and the CTI [12]. 

To be eligible for the CTI, and therefore eligible for our study, contacts were assessed by local PHUs as having high-risk exposure to a confirmed COVID-19 case. While there were some refinements to the definitions over the observation period, high-risk exposure was defined as close proximity to a confirmed COVID-19 case (less than 2 m) for at least 15 min or for multiple short periods of time without protective measures (e.g., masking, distancing, use of personal protective equipment). Contacts also had to be directly reachable, meaning that contacts in institutional settings who could only be reached through a staff member, such as a ward nurse, were ineligible. Therefore, patients in healthcare settings and residents of long-term care homes were ineligible. Some contacts submitted to the program were assessed as being ineligible and were sent back to PHUs after they were submitted to the CTI (e.g., if they were entered in error, their high-risk exposure was ruled out, or the case they were exposed to did not meet the case definition). PHUs were able to access contact information while contacts were assigned to the CTI, edit fields in the system during follow-up, and were able to remove contacts, which could impact whether contacts met our definition of completing follow-up. The process for PHUs to remove contacts, which did not reach 100% consistency due to limited capacity and training constraints, involved manually setting a field labelled ‘Disposition’ to a value labelled ‘Complete’. However, the reason for removal was typically not recorded. Our study excluded contacts who had their ‘Disposition’ field set to ‘Complete’ but otherwise met our definition of not completing the CTI program (i.e., completed final day call, became a case, or developed symptoms, as defined in Outcome Measures subsection below), as these contacts had discordant data on completing follow-up. Our study also excluded contacts with missing data on age, material resources, exposure setting, and preferred language, even if they were eligible and participated in the CTI. These data (including postal code used to determine material resources) were intended to be collected by PHUs, and updated by CTI callers if needed, but were sometimes missed due to time constraints and lack of capacity.

### Characteristics of contacts

Contact variables analyzed included: the contact’s age group, COVID-19 wave (sequentially numbered “waves” were the descriptors used during the initial years of the COVID-19 pandemic to partition the timeline according to fluctuations in confirmed case numbers) in which they became a contact, the quintile of their neighbourhood’s material resources, exposure setting (categorical field with an open text option), region of Ontario they live in, and preferred language. Previous studies have identified each of these factors as potentially affecting contact tracing adherence [7]. Callers asked contacts for their date of birth during the first call, although date of birth was not mandatory and contacts could proceed without providing this information. We determined the COVID-19 wave based on the date the contact was reported to the local PHU, and no data were missing for this variable. We used neighbourhood-level quintiles of material resources as a proxy measure of socio-economic status [14]. Exposure setting was entered by the local PHUs before submitting each contact to the CTI.

We examined 20–39 year olds as the reference age group, as this group comprised a large proportion of the overall respondents that did not include any contacts who required a proxy because they were younger than 13. We compared the following age groups to this group: 0–4, 5–11, 12–19, 40–59, 60–79, and 80+. These age groupings were chosen to align with other COVID-19 reports by Public Health Ontario [18, 19]. The timeline of the COVID-19 pandemic was divided into five distinct waves, which are shown in Fig. 1 [18]. Wave 1 (February 26, 2020 to August 31, 2020) of the pandemic was over before the CTI moved to the CCM system, so we used Wave 2 as the reference group and compared Waves 3, 4, and 5 to Wave 2 (Fig. 1). The reference group for material resources was quintile 1, neighbourhoods with the highest material resources. For exposure setting, we used schools as the reference group and compared the following exposure settings to schools: commercial establishments (e.g., restaurants), congregate living (e.g., retirement homes), medical (e.g., hospitals), workplaces (e.g., processing plants), and other settings (e.g., not specified). We identified the region of Ontario where contacts lived using a mandatory field in the CCM system that showed the local PHU leading the initial investigation. We used the Greater Toronto Area (GTA) as the reference group and compared the following regions of Ontario to the GTA: Eastern, Central East, Central West, South West, North West, and North East. These regions were adopted from Ontario’s COVID-19 wastewater surveillance program [20]. The GTA includes the city of Toronto, plus the suburban cities surrounding Toronto. The GTA comprises almost half of Ontario’s population and has a relatively high population density throughout most of the region. The remaining regions comprise smaller cities, small towns, and rural areas. Contacts reported their preferred language during the first phone call. English was the reference group, with all other languages as the comparison group.

### Outcome measures

Our two outcome measures were COVID-19 contacts who reported self-isolating and contacts who completed CTI follow-up. During the initial CTI call, contacts were instructed to self-isolate in their homes and only leave to get tested for COVID-19. For contacts who lived with others, they were also directed to self-isolate from household members for the duration of their isolation period (e.g., advised to stay in separate rooms as much as possible). Allowances were made in situations where it was impossible for contacts to self-isolate from certain people, such as a young child requiring contact with one parent or caregiver, or an individual of any age requiring a home visit by a care provider [21, 22]. 

During the study, the isolation period for COVID-19 contacts was shortened from 14 days to 10 days after exposure to align with changes to Ontario’s case and contact management guidelines. This change was implemented in the CTI on August 31, 2021. Exposure dates were recorded by local PHUs as the date no later than 24 h after a positive COVID-19 lab result for the contact’s corresponding case. The exposure date was considered ‘Day 1’ of the contact follow-up period. Contact tracers at PHUs and PHO did their best to reach each contact within 24 h of being submitted. On each subsequent call, contacts were asked if they had been self-isolating since their last call. If contacts answered ‘yes’ to this question during all subsequent calls, we included them in the self-isolating category. If they answered ‘no’ during one or more calls, we counted them as not self-isolating. Contacts received a call on the final day of their self-isolation or self-monitoring period. If contacts completed this call, we counted them as successfully completing follow-up. Contacts were withdrawn from the CTI by local PHUs if they became a COVID-19 case, and these were counted as completing follow-up. Contacts were sent back to local PHUs if they reported COVID-19 symptoms, and these were counted as completing follow-up as well. Completing the CTI, becoming a case, and developing symptoms were all captured through data generated in the CCM, which indicated that the CTI fulfilled its purpose. We counted contacts as not completing follow-up if they exited the program for another reasons. For example, some contacts did not answer after four to six call attempts or told callers they did not wish to complete the call or receive additional follow-up calls.

### Statistical analysis

We calculated the percentage of contacts in each of the categories for the exposure and outcome variables. We calculated unadjusted associations between each exposure variable and each of the two outcome variables, using separate bivariate logistic regression models for each exposure-outcome combination. The bivariate analyses produced unadjusted estimates for comparison with adjusted estimates from the multivariable analyses. We built two multivariable regression models, one for each outcome. Both multivariable models included all of the aforementioned exposure variables: age group, material resources, COVID-19 wave, exposure setting, region of Ontario, and preferred language. We performed forward selection to guide inclusion of variables for each model, where we added variables one by one and manually compared the following after each addition: changes in the point estimates and confidence intervals of variables already in the model, fit statistics including − 2 log likelihood and Akaike information criterion, and global tests of the null hypothesis. We also assessed the correlation between the independent variables. These assessments led to removal of a variable called type of exposure (e.g., workplace, healthcare) because it was highly correlated with the exposure setting variable. We conducted all analyses using SAS Enterprise Guide, version 8.2. We used *p* < 0.05 as the threshold for statistical significance, equivalent to odds ratio confidence intervals that do not overlap 1.0, and calculated adjusted p-values to correct for multiple comparisons (26 comparisons for each model) using the Benjamini-Hochberg method. This method was chosen over the Bonferroni method because of concerns the Bonferroni method has poor statistical power [23]. 

### Sensitivity analyses

We performed five sensitivity analyses using several different contact eligibility criteria. We analyzed both outcomes in a sample population where nobody was removed due to missing data; instead, a separate category was created for the missing responses. For the self-isolation outcome only, we excluded anyone who did not receive a subsequent call, in which they were asked if they had been self-isolating. For the completed follow-up outcome only, we excluded anyone who did not complete the final call, but at some point during follow-up was flagged as having a wrong number or was returned to the heath unit due to missing data or failure to meet the CTI’s eligibility requirements. For the completed follow-up outcome only, we also kept those who were excluded from the main analysis because they had their ‘Disposition’ field set to ‘Complete’, despite otherwise meeting our definition of completing follow-up, because the reasons these were set to ‘Complete’ were generally unknown.

## Results

### Population characteristics

A total of 263,849 high-risk COVID-19 contacts in Ontario were submitted via the CTI’s CCM system between September 1, 2020 and December 31, 2021. We excluded 24,349 (9.2%) of these contacts because they had the ‘Disposition’ field set to ‘Complete’ but did not have the final Contact Tracing Complete task created in the CCM system, leaving 239,500 contacts without this discordant information. We excluded an additional 38,446 (14.6%) contacts because they were missing age, another 40,729 (15.4%) because they were missing data on material resources, another 13,180 (5.0%) because they were missing exposure setting data, and another 16,890 (6.4%) because they were missing preferred language. Of the 40,729 contacts missing data on material resources, 44.6% of were missing a postal code in the CCM system, while the rest were matched to locations in the Postal Code Conversion File that did not have a match to material resources data. Our final sample size for the main analysis was 130,255.

Table 1 shows the number and percentage of respondents in each of the exposure categories and each of the outcome categories. The 80 years and older age group had the smallest proportion of contacts, representing only 1.3% of all contacts. Compared to the other three waves included in our study, Wave 5 had the fewest contacts by far, with only 1.9% of all contacts. People were spread fairly evenly across the material resources quintiles, although the quintile with the least resources, quintile 5, had the smallest percentage at 16.5%. More contacts were exposed in schools (37.2% of included contacts) or congregate living settings (31.1% of included contacts) than other types of settings. The GTA was the region with the most contacts (37.4% of included contacts), which was expected because the GTA contains almost half of Ontario’s population. Finally, the vast majority of contacts had English as their preferred language (98.7%). A study by Chambers et al. examined the language requirements of CTI contacts more closely and identified the following as the top five languages requested for interpretation: French, Arabic, Spanish, Punjabi, and Mandarin [24]. 

**Table 1:** Characteristics of Contact Tracing Initiative participants included in study sample (*N* = 130,255)

Variable	Level	*N*	%
Age Group, years	0 to 4	7,170	5.5
	5 to 11	33,239	25.5
	12 to 19	30,647	23.5
	20 to 39	27,065	20.8
	40 to 59	21,290	16.3
	60 to 79	9,121	7.0
	80+	1,723	1.3
Material Resources	Quintile 1	25,435	19.5
	Quintile 2	30,614	23.5
	Quintile 3	29,217	22.4
	Quintile 4	23,463	18.0
	Quintile 5	21,526	16.5
COVID-19 Wave	2 (01/09/2020-28/02/2021)	31,787	24.4
	3 (01/03/2021-31/07/2021)	39,734	30.5
	4 (01/08/2021-14/12/2021)	56,321	43.2
	5 (15/12/2021-28/02/2022)	2,413	1.9
Exposure Setting	Commercial Establishment	4,342	3.3
	Congregate Living	40,526	31.1
	Medical	3,856	3.0
	Other^a^	26,617	20.4
	School	48,473	37.2
	Workplace	6,441	4.9
Region	Central East	7,125	5.5
	Central West	40,790	31.3
	Eastern	9,136	7.0
	Greater Toronto Area (GTA)	48,719	37.4
	North East	7,708	5.9
	North West	1,582	1.2
	South West	15,195	11.7
Preferred Language	English	129,093	99.1
	Non-English	1,162	0.9
Completed follow-up	Yes	116,506	89.4
	No	13,749	10.6
Reported self-isolating on follow-up calls	Yes	128,620	98.7
	No	1,635	1.3

^a^’Other’ exposure settings include child care centres, recreation/community centres, short-term accommodations, and settings that did not fit in other categories

### Outcome: Self-isolating

Only 1.3% of the study population reported that they had not self-isolated during at least one follow-up call (Table 1), 7.3% of whom did not end up completing follow-up. In the multivariable analysis of self-isolating, 0–4 and 5–11 year olds had significantly lower odds of self-isolating than 20–29 year olds (adjusted odds ratio (AOR) [95% confidence interval (95% CI)] = 0.60 [0.48–0.75] for ages 0–4 and 0.57 [0.48–0.67] for ages 5–11), while the other age groups showed much smaller differences that were not significantly different (Table 2). People living in areas within the middle quintile of material resources (quintile 3) had lower odds of self-isolating than people in the quintile with the most resources (quintile 1) (AOR [95% CI] = 0.84 [0.72–0.98]), although this became non-significant after adjusting for multiple comparisons. None of the other statistically significant estimates in the multivariable analysis looking at adherence to self-isolation became non-significant after adjusting for multiple comparisons (Table 2). Differences in self-isolating for people in the other three material resources quintiles were slight and were not statistically significant. Compared to Wave 2, CTI participants had significantly lower odds of self-isolating during Wave 3 (AOR [95% CI] = 0.73 [0.64–0.83]), but significantly higher odds in Waves 4 and 5 (AOR [95% CI] = 1.49 [1.30–1.71] for Wave 4 and 2.41 [1.41–4.13] for Wave 5). Compared to schools, contacts who were exposed in different categories of settings had significantly lower odds of self-isolating, although the differences were significant only for congregate living (AOR [95% CI] = 0.82 [0.71–0.95]) and other (AOR [95% CI] = 0.82 [0.71–0.95]) settings. The North East and North West regions of Ontario had significantly lower odds of self-isolating than the GTA (AOR [95% CI] = 0.78 [0.64–0.95] for North East and 0.64 [0.45–0.92] for North West), while the other regions showed no significant differences from the GTA. Finally, contacts who preferred speaking in a language other than English had significantly lower odds of self-isolating (AOR [95% CI] = 0.57 [0.38–0.84]). Most confidence intervals were wide and those for exposure setting and region were close to the threshold of statistical significance.

**Table 2:** Unadjusted and adjusted odds of self-isolating according to various characteristics (*N* = 130,255)

Comparison	Unadjusted estimates from separate bivariate analyses (odds ratio & 95% confidence interval)	Adjusted^a^ estimates from one multivariable analysis (odds ratio & 95% confidence interval)
Age Group (years) 0 to 4	**0.67 (0.54**,** 0.83)**	**0.60 (0.48**,** 0.75)**
5 to 11	**0.72 (0.62**,** 0.83)**	**0.57 (0.48**,** 0.67)**
12 to 19	1.03 (0.88, 1.20)	0.86 (0.72, 1.02)
20 to 39	Reference	Reference
40 to 59	0.89 (0.76, 1.06)	0.88 (0.75, 1.04)
60 to 79	0.91 (0.73, 1.13)	0.92 (0.74, 1.15)
80+	0.85 (0.55, 1.32)	0.93 (0.60, 1.46)
Material Resources Quintile 1	Reference	Reference
Quintile 2	0.90 (0.77, 1.05)	0.90 (0.77, 1.04)
Quintile 3	**0.84 (0.72**,** 0.98)**	**0.84 (0.72**,** 0.98)**
Quintile 4	0.95 (0.80, 1.12)	0.96 (0.81, 1.13)
Quintile 5	0.95 (0.80, 1.12)	0.95 (0.80, 1.12)
COVID-19 Wave Wave 2 (01/09/2020-28/02/2021)	Reference	Reference
Wave 3 (01/03/2021-31/07/2021)	**0.72 (0.64**,** 0.81)**	**0.73 (0.64**,** 0.83)**
Wave 4 (01/08/2021-14/12/2021)	**1.40 (1.22**,** 1.59)**	**1.49 (1.30**,** 1.71)**
Wave 5 (15/12/2021-28/02/2022)	**2.21 (1.30**,** 3.77)**	**2.41 (1.41**,** 4.13)**
Exposure Setting School	Reference	Reference
Commercial Establishment	0.96 (0.73, 1.28)	0.80 (0.59, 1.08)
Congregate	0.92 (0.82, 1.04)	**0.82 (0.71**,** 0.95)**
Medical	0.91 (0.68, 1.21)	0.77 (0.56, 1.06)
Other†	**0.87 (0.76**,** 0.99)**	**0.82 (0.71**,** 0.95)**
Workplace	0.87 (0.69, 1.09)	0.78 (0.61, 1.01)
Region of Ontario Greater Toronto Area (GTA)	Reference	Reference
Central East	0.96 (0.77, 1.19)	0.86 (0.69, 1.07)
Central West	**1.22 (1.08**,** 1.38)**	1.05 (0.93, 1.19)
Eastern	1.10 (0.90, 1.34)	0.98 (0.80, 1.21)
North East	0.85 (0.70, 1.03)	**0.78 (0.64**,** 0.95)**
North West	**0.63 (0.45**,** 0.90)**	**0.64 (0.45**,** 0.92)**
South West	1.15 (0.97, 1.36)	1.13 (0.96, 1.34)
Preferred Language English	Reference	Reference
Non-English	**0.55 (0.37**,** 0.82)**	**0.57 (0.38**,** 0.84)**

^a^Variables included in adjusted model: age group, material resources, COVID-19 wave, exposure setting, region of Ontario, and preferred language. Statistically significant estimates at *p* < 0.05 indicated in bold. †’Other’ exposure settings include child care centres, recreation/community centres, short-term accommodations, and settings that did not fit into the other categories.

### Outcome: completed Follow-up

Roughly 90% of the study population met our definition of completing follow-up (Table 1). Of these, 8% became a case and just over one quarter reported symptoms but were not recorded as becoming a case, with the remaining completing the final call. In the multivariable analysis of completing follow-up, compared to the reference group (ages 20–39), the youngest (ages 0–4) and all older (40–59, 60–79, and 80+) age groups had significantly higher odds of completing follow-up (AOR [95% CI] = 1.27 [1.16–1.39] for ages 0–4, 1.28 [1.21–1.36] for ages 40–59, 1.34 [1.24–1.46] for ages 60–79, and 1.30 [1.10–1.54] for ages 80+), while the other age groups had no significant differences (Table 3). Contacts living in material resources quintiles 2–5, with higher quintiles representing areas with fewer material resources, had significantly lower odds of completing follow-up than those in quintile 1 (AOR [95% CI] = 0.89 [0.84–0.94] for quintile 2, 0.78 [0.73–0.82] for quintile 3, 0.70 [0.66–0.74] for quintile 4, and 0.57 [0.54–0.60] for quintile 5). This relationship followed a gradient where those living in neighbourhoods with successively fewer material resources were less and less likely to complete follow-up. Compared to Wave 2, contacts from Waves 3 and 4 had significantly higher odds of completing follow-up (AOR [95% CI] = 1.05 [1.00-1.10 for Wave 3 and 1.28 [1.23–1.35] for Wave 4), while contacts from Wave 5 had significantly lower odds (AOR [95% CI] = 0.63 [0.56–0.71]). Compared to schools, people exposed in commercial establishments, medical settings, or workplaces had significantly lower odds of completing follow-up (AOR [95% CI] = 0.90 [0.81–0.99] for commercial establishments, 0.82 [0.73–0.92] for medical settings, and 0.88 [0.81–0.97] for workplaces), while people exposed in congregate living or other settings had significantly higher odds (AOR [95% CI] = 1.06 [1.01–1.12] for congregate living and 1.07 [1.02–1.13] for other settings); however, all confidence intervals were close to the threshold of statistical significance. Compared to the GTA, all other regions of Ontario had significantly higher odds of completing follow-up (AOR [95% CI] = 1.42 [1.30–1.54] for Central East, 1.19 [1.14–1.24] for Central West, 1.23 [1.14–1.33] for Eastern, 1.31 [1.21–1.43] for North East, 1.58 [1.32–1.88] for North West, and 1.91 [1.78–2.04] for South West). Lastly, people who preferred speaking in a language other than English had lower odds of completing follow-up, but this difference was not statistically significant (AOR [95% CI] = 0.89 [0.75–1.06). None of the statistically significant estimates of completing follow-up became non-significant after adjusting for multiple comparisons.

**Table 3:** Unadjusted and adjusted odds of completing follow-up according to various characteristics (*N* = 130,255)

Comparison	Unadjusted estimates from separate bivariate analyses (odds ratio & 95% confidence interval)	Adjusted^a^ estimates from one multivariable analysis (odds ratio & 95% confidence interval)
Age Group (years) 0 to 4	**1.34 (1.23**,** 1.47)**	**1.27 (1.16**,** 1.39)**
5 to 11	**1.11 (1.05**,** 1.17)**	1.04 (0.98, 1.10)
12 to 19	**1.06 (1.01**,** 1.11)**	0.98 (0.93, 1.04)
20 to 39	Reference	Reference
40 to 59	**1.31 (1.23**,** 1.39)**	**1.28 (1.21**,** 1.36)**
60 to 79	**1.35 (1.24**,** 1.46)**	**1.34 (1.24**,** 1.46)**
80+	**1.21 (1.03**,** 1.42)**	**1.30 (1.10**,** 1.54)**
Material Resources Quintile 1	Reference	Reference
Quintile 2	**0.89 (0.84**,** 0.94)**	**0.89 (0.84**,** 0.94)**
Quintile 3	**0.80 (0.75**,** 0.84)**	**0.78 (0.73**,** 0.82)**
Quintile 4	**0.72 (0.68**,** 0.77)**	**0.70 (0.66**,** 0.74)**
Quintile 5	**0.60 (0.57**,** 0.64)**	**0.57 (0.54**,** 0.60)**
COVID-19 Wave Wave 2 (01/09/2020-28/02/2021)	Reference	Reference
Wave 3 (01/03/2021-31/07/2021)	**1.05 (1.01**,** 1.10)**	**1.05 (1.00**,** 1.10)**
Wave 4 (01/08/2021-14/12/2021)	**1.29 (1.24**,** 1.35)**	**1.28 (1.23**,** 1.35)**
Wave 5 (15/12/2021-28/02/2022)	**0.64 (0.58**,** 0.72)**	**0.63 (0.56**,** 0.71)**
Exposure Setting School	Reference	Reference
Commercial Establishment	0.92 (0.84, 1.02)	**0.90 (0.81**,** 0.99)**
Congregate	**1.06 (1.01**,** 1.10)**	**1.06 (1.01**,** 1.12)**
Medical	0.91 (0.82, 1.00)	**0.82 (0.73**,** 0.92)**
Other†	**1.17 (1.11**,** 1.23)**	**1.07 (1.02**,** 1.13)**
Workplace	**0.90 (0.83**,** 0.98)**	**0.88 (0.81**,** 0.97)**
Region of Ontario Greater Toronto Area (GTA)	Reference	Reference
Central East	**1.38 (1.27**,** 1.50)**	**1.42 (1.30**,** 1.54)**
Central West	**1.13 (1.08**,** 1.17)**	**1.19 (1.14**,** 1.24)**
Eastern	**1.16 (1.08**,** 1.25)**	**1.23 (1.14**,** 1.33)**
North East	**1.28 (1.18**,** 1.39)**	**1.31 (1.21**,** 1.43)**
North West	**1.39 (1.16**,** 1.65)**	**1.58 (1.32**,** 1.88)**
South West	**1.77 (1.66**,** 1.90)**	**1.91 (1.78**,** 2.04)**
Preferred Language English	Reference	Reference
Non-English	0.85 (0.71, 1.01)	0.89 (0.75, 1.06)

^a^Variables included in adjusted model: age group, material resources, COVID-19 wave, exposure setting, region of Ontario, and preferred language. Statistically significant estimates at p < 0.05 indicated in bold. †’Other’ exposure settings include child care centres, recreation/community centres, short-term accommodations, and settings that did not fit into the other categories.

### Sensitivity analyses

The two sensitivity analyses performed for the self-isolating outcome, one where contacts were excluded if they didn’t have a second call from the CTI and another where contacts missing data were kept in the analysis, showed largely similar results as the main analysis of self-isolating (Table 2 & Supplementary Tables 1–2). The only large differences in effect sizes were for waves, where the odds of self-isolating in later waves compared with Wave 2 showed sizeable attenuations in both sensitivity analyses of the self-isolating outcome. In two of the three sensitivity analyses for the completing follow-up outcome, all estimates had similar magnitudes of association as the main analysis: that is, the analysis where contacts missing data were kept, and the analysis where contacts were excluded if they did not complete the CTI but also had a wrong number or were returned to local PHUs due to missing/incorrect information or ineligibility (Table 3 & Supplementary Tables 3–4). However, there were a number of marked differences in the third sensitivity analysis of the completing follow-up outcome, which was the analysis where contacts were kept if they had their disposition field set to complete but did not otherwise meet our definition of completing follow-up. There were large changes in the magnitude of effect accompanied by a reversal of the direction of effect for certain comparisons of age, COVID-19 wave, exposure setting, and region (Table 3 & Supplementary Table 5). The most stable estimates in this sensitivity analysis were for material resources, which saw a slight attenuation of effect, but still significantly lower odds of completing follow-up among contacts in areas with fewer resources and still a gradient of decreasing odds in each quintile with successively fewer resources. For both outcomes, there were several estimates that were statistically significant in the main analysis that were no longer significant in the sensitivity analyses, and vice versa; however, each of these estimates was close to the threshold of statistical significance at *p* = 0.05. There were also estimates that were in the opposite direction in the sensitivity analyses, but each of these was a non-significant effect in both the main and sensitivity analyses.

## Discussion

This study examined reported self-isolation and completion of a COVID-19 contact tracing program using data collected directly from the operations of a contact tracing program. Our results show that contacts were less likely to self-isolate if they were young children (0–4 and 5–11 year of age), spoke a language other than English, or were exposed during earlier waves of the pandemic; however, variability around these estimates was high, likely because only 1.3% of contacts said they were not self-isolating. The pattern of self-isolating was less clear in relation to material resources, exposure setting, and region of residence. Our results also show that contacts were less likely to complete follow-up if they lived in areas with fewer material resources, were in an older age group, or if they lived in the GTA. The pattern of association for completing follow-up in relation to COVID-19 wave, exposure setting, and preferred language was less clear.

The most robust finding in our study was that contacts living in quintiles with fewer material resources had lower odds of completing follow-up. This relationship also held in each of the sensitivity analyses. Many people in areas with fewer resources face inequities that may impact their ability to complete follow-up, such as greater exposure to poor working conditions, insecure housing, greater barriers to accessing healthcare, and chronic stress from everyday material deprivation [25]. Such inequities also relate to lower levels of trust in health authorities among people in areas with fewer material resources [26, 27]. Our results show a gradient in rates of those completing CTI across quintiles of material resources that is congruent with Ontario data showing a material resources gradient in COVID-19 cases, hospital admissions, intensive care unit admissions, and deaths, all of which were higher in areas with lower levels of material resources [19]. 

Regarding the self-isolating outcome, our results showed that 0–4 and 5–11 year olds were less likely than the other age groups to have reported, by proxy, that they self-isolated. There are a number of practical challenges in adhering to self-isolation practices for these age groups [28]. While allowances were made for one caregiver to self-isolate with a child who was a contact, it can be difficult for younger contacts to self-isolate from other household members because there may not be enough space in the home to self-isolate from other family members, or the caregiver may also be a caregiver for other household members who are not contacts. The lower rate of self-isolation and completion of follow-up among people who preferred to speak in a language other than English is likely due to many factors, one of which could be the added step of initiating and using interpretation services, lengthening call times. It should also be noted that the low percentage of people requesting a call in a language other than English may be due to interpretation services not always being requested by the contact or recognized by the caller, languages other than English not being recorded by the caller, or the use of a proxy respondent with better knowledge of English. Lastly, the results showing higher odds of self-isolating in later COVID-19 waves, while varying in strength across the sensitivity analyses, may point to a true increase in self-isolating that could be due to various factors, such as shortening of the required self-isolation time during the study period, shifts to different types of contacts assigned to the CTI who could more easily self-isolate or were more risk averse and amenable to self-isolating, or even a greater tendency to misreport self-isolation as COVID-19 fatigue set in.

Few published studies have used real-time data generated during calls to COVID-19 to examine adherence to self-isolation guidelines and completion of contact follow-up as indicators of contact tracing effectiveness [10]. At the time of writing, we found only one published study that used data collected during calls and examined indicators similar to ours. Herrero et al. conducted a study in Catalonia, Spain where they retrospectively analyzed data collected during their COVID-19 contact tracing program [29]. However, they estimated the percentage ’accepting follow-up’, assessed as the percentage who did not have data errors, invalid ID, no answer, or refusal. This metric was different from that used in our study, so results are not comparable. There are several published studies that deployed a survey to a sample of contacts, rather than using data collected during contact follow-up calls, and therefore have higher risk of certain biases, such as recall bias or non-response bias [7, 30–32]. On the other hand, our study may have a higher risk of social desirability bias, as contacts may have feared repercussions if they reported non-adherence to self-isolation while they were supposed to be self-isolating. Nevertheless, the 98.7% of contacts who reported adherence to self-isolation in our study was close to the upper end of the 78%−94% range of contact adherence to self-isolation in studies included in a systematic review by Smith et al. [7] This systematic review also found little evidence that self-isolation adherence was associated with sociodemographic factors, including older age, earlier timepoint in the pandemic, living in less deprived areas, or living in urban versus rural areas. Our study did find that younger contacts were less likely to self-isolate, possibly because we included younger age groups than most studies included in the Smith review. Our study also showed contacts were less likely to self-isolate during earlier waves of the pandemic, although variability around these estimates was high.

Future research should leverage data collected by systems in other jurisdictions during the unprecedented scale-up of contact tracing during the COVID-19 pandemic. In addition to increasing the generalizability of results, research in other jurisdictions can examine additional factors associated with adherence to self-isolation and contact follow-up (e.g. occupation, substance use, etc.), ideally measured at the individual level, thereby expanding knowledge on what factors can impact contact tracing outcomes and how to target program resources.

A major strength of this study is the large sample size, which provided enough power for us to analyze multiple exposure variables in multivariable models. Another strength is the diverse, Ontario-wide study population that covers all ages, which enhances the generalizability of the results. On the other hand, limitations included reduced generalizability because we did not have a random study sample that was representative of all COVID-19 contacts in Ontario. Another limitation is possible outcome misclassification. Some contacts may have inaccurately responded to the question about self-isolation, for example, by saying they were self-isolating when they were not, and we counted these contacts as self-isolating. Likewise, some contacts may have stopped responding to calls after they stopped self-isolating, so would have been counted as not completing the CTI rather than not self-isolating. There may also have been data entry errors by the callers that resulted in misclassification for both outcomes. Outcome misclassification may have differed across age groups, material resources quintiles, COVID-19 waves, regions, and exposure settings, resulting in over or under-estimation of the associations with the two outcomes. While Ontario had specific data entry requirements for COVID-19 cases, there were no specific requirements for high-risk contacts, and there may have been procedural differences between different PHUs and CTI staff. Therefore, only CCM data entry fields (name, phone number, PHU responsible) required from a system perspective were consistently entered. This limited the number of contacts for whom we could analyze specific characteristics (e.g. contacts missing postal code were removed from the analysis). There may have been individuals who exited the CTI, then became a contact again at a later date and re-entered the CTI. We removed duplicate contact investigations that had overlapping time periods for the same individual. However, we were unable to identify individuals who may have entered the CTI at multiple distinct time periods, therefore, we were unable to account for the correlation between separate contact investigations for the same individual. This may have resulted in an underestimation of the confidence intervals around the estimates. An additional limitation is that there may be confounding variables that we were not able to include, resulting in residual confounding.

Overall, this study demonstrates utility of analyzing contact tracing data for applied research. The call scripts and data entry requirements were standardized across all calls made by the provincial CTI, which created an opportunity to analyze a large province-wide sample of contacts that had standardized data collection. While the degree of standardization was necessary for province-wide reporting, it presented challenges in adapting to unique needs of individual PHUs and the timeliness of modifications to the scripts. The evolving guidance around contact tracing and self-isolation made collection of consistent measures more challenging. Changes in public health measures, case and contact management guidelines, and individuals’ behaviours must also be taken into account when interpreting results. Nonetheless, Ontario’s province-wide CTI not only provided support to local PHUs and provided the ability to rapidly scale-up call volume, it also enabled standardized messaging, caller training, and data collection that facilitated comparisons of contact tracing indicators across populations, regions, and time periods.

## Conclusions

Our study findings point to several characteristics that may influence adherence to self-isolation guidelines and contact follow-up. For instance, people in areas with fewer material resources, and spoken language preferences other than English, may require additional supports to assist in completion of follow-up. If future research corroborates these findings with targeted interventions, future contact tracing programs can adapt their strategies for these populations and for these exposure settings.

## Supplementary Information

Supplementary Material 1

## Data Availability

The data that support the findings of this study are restricted to protect the privacy of the study population and ensure the confidentiality of their data. Therefore, these data are not publicly available and are not available upon request to the authors.

## Abbreviations

CCM: Case and Contact Management system
CTI: Contact Tracing Initiative
GTA: Greater Toronto Area
PHUs: Public Health Units
