# Supplementary material for: Factors associated with self-isolating and completing a contact follow-up program: a retrospective analysis of Ontario, Canada’s COVID-19 contact tracing initiative
Source: BMC Public Health. 2025 Nov 26;25:4163. doi: 10.1186/s12889-025-24737-2 (PMC12659302; doi:10.1186/s12889-025-24737-2)
Supplement: Supplementary file 1 — Supplementary Material 1 [file 12889_2025_24737_MOESM1_ESM.docx]

# Supplementary Appendix

Description of Public Health Ontario’s Contact Tracing Initiative and Case and Contact Management System:

The CTI was started in March 2020, and in September and October of 2020 was transitioned to a new provincial Case and Contact Management (CCM) system. The CCM provided a unified system where each local PHU could enter information about the contacts and assign the contacts to the CTI. It also enabled standardized information and data collection among CTI callers with embedded call scripts and questions.^1^

If individuals required interpretation services, the caller could access interpretation services providers to provide simultaneous interpretation in the contact’s preferred language. If individuals were younger than age 13, proxy interviewees were required to answer questions on their behalf. Older individuals could designate proxy interviewees upon request to the caller. At the onset of the pandemic, guidance required all high-risk contacts to self-isolate; however, who was required to self-isolate, when, and for how long, varied throughout different phases of the pandemic. For example, during Wave 4, contacts that reported being fully vaccinated and asymptomatic were not required to isolate and were simply directed to monitor their symptoms. During the first call, callers informed contacts that they should self-isolate in their homes for a specified period of time. During subsequent calls, callers asked contacts if they had adhered to this request to self-isolate and recorded their responses in the CCM. If contacts stated that they did not wish to receive additional follow-up calls, callers recorded this in the CCM as well. Callers also recorded whether a call was not answered and if four to six attempts were unanswered the contact was marked as lost to follow-up. Contact records in the CCM were assigned back to the PHU after they completed the program, refused to participate, were lost to follow-up, or became symptomatic. The CCM automatically generated ‘tasks’ when the caller entered information about the contact and the contact’s progression through the CTI. For instance, the system generated a ‘Contact Tracing Complete’ task upon successful completion of the final follow-up call.

Contacts are only included in this study if they were referred to the CTI after local PHUs transitioned to using the CCM system (September/October 2020). Contacts referred after December 31, 2021 were excluded, because at this time Ontario’s contact tracing guidance no longer required follow-up of most contacts, due to reprioritization of resources during the spread of the Omicron variant.^2^

**Supplementary table 1: Unadjusted and adjusted odds of self-isolating according to various characteristics, including participants missing data (N=239,500)**

| Comparison | Unadjusted estimates from separate bivariate analyses | Adjusted*  estimates from one multivariable analysis |
| --- | --- | --- |
| Age Group (years)  0 to 4 | **0.65 (0.54, 0.77)** | **0.59 (0.50, 0.71)** |
| 5 to 11 | **0.74 (0.66, 0.83)** | **0.59 (0.52, 0.67)** |
| 12 to 19 | 1.01 (0.89, 1.15) | **0.86 (0.75, 0.99)** |
| 20 to 39 | Reference | Reference |
| 40 to 59 | 0.91 (0.79, 1.04) | 0.89 (0.78, 1.03) |
| 60 to 79 | 0.97 (0.80, 1.16) | 0.98 (0.82, 1.19) |
| 80+ | 0.78 (0.55, 1.10) | 0.85 (0.60, 1.21) |
| Missing | 0.88 (0.77, 1.00) | 0.90 (0.78, 1.03) |
| Material Resources  Quintile 1 | Reference | Reference |
| Quintile 2 | 0.90 (0.79, 1.03) | 0.91 (0.79, 1.03) |
| Quintile 3 | 0.89 (0.78, 1.02) | 0.90 (0.79, 1.03) |
| Quintile 4 | 0.93 (0.81, 1.07) | 0.95 (0.82, 1.09) |
| Quintile 5 | 0.92 (0.79, 1.06) | 0.93 (0.80, 1.07) |
| Missing | 0.98 (0.87, 1.11) | 0.96 (0.85, 1.09) |
| COVID-19 Wave  Wave 2 (01/09/2020-28/02/2021) | Reference | Reference |
| Wave 3 (01/03/2021-31/07/2021) | **0.78 (0.72, 0.86)** | **0.80 (0.73, 0.87)** |
| Wave 4 (01/08/2021-14/12/2021) | **1.46 (1.32, 1.60)** | **1.57 (1.42, 1.74)** |
| Wave 5 (15/12/2021-28/02/2022) | **1.97 (1.32, 2.94)** | **2.09 (1.39, 3.12)** |
| Exposure Setting  School | Reference | Reference |
| Commercial Establishment | 0.93 (0.75, 1.14) | **0.77 (0.61, 0.96)** |
| Congregate | 0.92 (0.84, 1.02) | **0.82 (0.73, 0.92)** |
| Medical | 0.82 (0.66, 1.03) | **0.70 (0.55, 0.89)** |
| Other | **0.87 (0.79, 0.96)** | **0.81 (0.73, 0.90)** |
| Workplace | 0.97 (0.81, 1.17) | 0.86 (0.70, 1.05) |
| Missing | **0.80 (0.71, 0.91)** | 0.84 (0.73, 0.97) |
| Region of Ontario  Greater Toronto Area (GTA) | Reference | Reference |
| Central East | 1.00 (0.85, 1.17) | 0.88 (0.75, 1.03) |
| Central West | **1.24 (1.13, 1.35)** | 1.08 (0.98, 1.18) |
| Eastern | 1.16 (1.00, 1.36) | 1.00 (0.86, 1.18) |
| North East | **0.79 (0.69, 0.90)** | **0.70 (0.61, 0.80)** |
| North West | **0.69 (0.50, 0.93)** | **0.70 (0.51, 0.95)** |
| South West | 1.05 (0.93, 1.19) | 1.00 (0.88, 1.14) |
| Preferred Language  English | Reference | Reference |
| Non-English | 0.77 (0.54, 1.10) | 0.79 (0.55, 1.14) |
| Missing | 0.94 (0.85, 1.05) | 0.97 (0.86, 1.08) |

*Variables included in adjusted model: age group, material resources, COVID-19 wave, exposure setting, region of Ontario, and preferred language. Statistically significant estimates at p < 0.05 indicated in bold.

**Supplementary table 2: Unadjusted and adjusted odds of self-isolating according to various characteristics, among participants who completed one or more subsequent calls (N=99,322)**

| Comparison | Unadjusted estimates from separate bivariate analyses | Adjusted*  estimates from one multivariable analysis |
| --- | --- | --- |
| Age Group (years)  0 to 4 | **0.67 (0.54, 0.83)** | **0.64 (0.51, 0.80)** |
| 5 to 11 | **0.79 (0.68, 0.91)** | **0.63 (0.53, 0.74)** |
| 12 to 19 | 1.11 (0.95, 1.31) | 0.91 (0.76, 1.08) |
| 20 to 39 | Reference | Reference |
| 40 to 59 | 0.91 (0.77, 1.08) | 0.90 (0.76, 1.07) |
| 60 to 79 | 0.92 (0.74, 1.16) | 0.94 (0.75, 1.18) |
| 80+ | 0.83 (0.53, 1.28) | 0.91 (0.59, 1.43) |
| Material Resources  Quintile 1 | Reference | Reference |
| Quintile 2 | 0.87 (0.75, 1.01) | 0.87 (0.75, 1.02) |
| Quintile 3 | **0.81 (0.69, 0.94)** | **0.81 (0.70, 0.95)** |
| Quintile 4 | 0.91 (0.77, 1.07) | 0.92 (0.78, 1.09) |
| Quintile 5 | 0.88 (0.74, 1.04) | 0.88 (0.74, 1.04) |
| COVID-19 Wave  Wave 2 (01/09/2020-28/02/2021) | Reference | Reference |
| Wave 3 (01/03/2021-31/07/2021) | **0.65 (0.57, 0.74)** | **0.66 (0.58, 0.75)** |
| Wave 4 (01/08/2021-14/12/2021) | **1.18 (1.04, 1.35)** | **1.23 (1.07, 1.41)** |
| Wave 5 (15/12/2021-28/02/2022) | 1.68 (0.98, 2.86) | **1.75 (1.02, 3.01)** |
| Exposure Setting  School | Reference | Reference |
| Commercial Establishment | 0.89 (0.67, 1.20) | 0.79 (0.58, 1.07) |
| Congregate | **0.85 (0.75, 0.96)** | **0.79 (0.68, 0.91)** |
| Medical | 0.83 (0.62, 1.11) | 0.75 (0.54, 1.03) |
| Other | **0.79 (0.69, 0.91)** | **0.79 (0.68, 0.92)** |
| Workplace | 0.80 (0.64, 1.01) | 0.78 (0.60, 1.01) |
| Region of Ontario  Greater Toronto Area (GTA) | Reference | Reference |
| Central East | 1.01 (0.82, 1.26) | 0.91 (0.73, 1.13) |
| Central West | **1.29 (1.14, 1.46)** | **1.13 (1.00, 1.29)** |
| Eastern | 1.19 (0.97, 1.46) | 1.10 (0.89, 1.37) |
| North East | 0.83 (0.68, 1.01) | **0.77 (0.63, 0.95)** |
| North West | **0.69 (0.48, 0.99)** | 0.72 (0.50, 1.04) |
| South West | **1.22 (1.03, 1.44)** | **1.22 (1.03, 1.45)** |
| Preferred Language  English | Reference | Reference |
| Non-English | **0.53 (0.36, 0.78)** | **0.53 (0.36, 0.79)** |

*Variables included in adjusted model: age group, material resources, COVID-19 wave, exposure setting, region of Ontario, and preferred language. Statistically significant estimates at p < 0.05 indicated in bold.

**Supplementary table 3: Unadjusted and adjusted odds of completing follow-up according to various characteristics, including participants missing data (N=239,500)**

| Comparison | Unadjusted estimates from separate bivariate analyses | Adjusted*  estimates from one multivariable analysis |
| --- | --- | --- |
| Age Group (years)  0 to 4 | **1.28 (1.19, 1.37)** | **1.30 (1.21, 1.39)** |
| 5 to 11 | **1.07 (1.02, 1.11)** | **1.09 (1.04, 1.14)** |
| 12 to 19 | 0.97 (0.93, 1.01) | 0.97 (0.93, 1.01) |
| 20 to 39 | Reference | Reference |
| 40 to 59 | **1.33 (1.26, 1.40)** | **1.31 (1.25, 1.38)** |
| 60 to 79 | **1.36 (1.27, 1.46)** | **1.36 (1.27, 1.46)** |
| 80+ | 1.11 (0.97, 1.27) | **1.20 (1.05, 1.38)** |
| Missing | **0.46 (0.44, 0.48)** | **0.48 (0.46, 0.50)** |
| Material Resources  Quintile 1 | Reference | Reference |
| Quintile 2 | **0.89 (0.85, 0.94)** | **0.90 (0.86, 0.95)** |
| Quintile 3 | **0.80 (0.76, 0.84)** | **0.79 (0.75, 0.83)** |
| Quintile 4 | **0.71 (0.67, 0.74)** | **0.69 (0.66, 0.73)** |
| Quintile 5 | **0.62 (0.59, 0.65)** | **0.59 (0.56, 0.62)** |
| Missing | **0.53 (0.51, 0.55)** | **0.72 (0.69, 0.75)** |
| COVID-19 Wave  Wave 2 (01/09/2020-28/02/2021) | Reference | Reference |
| Wave 3 (01/03/2021-31/07/2021) | 0.98 (0.95, 1.01) | **1.04 (1.01, 1.08)** |
| Wave 4 (01/08/2021-14/12/2021) | **1.21 (1.17, 1.25)** | **1.16 (1.12, 1.20)** |
| Wave 5 (15/12/2021-28/02/2022) | **0.67 (0.62, 0.74)** | **0.58 (0.53, 0.64)** |
| Exposure Setting  School | Reference | Reference |
| Commercial Establishment | **0.83 (0.78, 0.89)** | 0.94 (0.87, 1.01) |
| Congregate | 0.98 (0.95, 1.01) | 1.03 (0.99, 1.07) |
| Medical | **0.83 (0.77, 0.90)** | **0.79 (0.73, 0.86)** |
| Other | 0.99 (0.95, 1.02) | **1.04 (1.00, 1.08)** |
| Workplace | **0.85 (0.80, 0.90)** | 0.94 (0.88, 1.00) |
| Missing | **0.86 (0.83, 0.90)** | **1.06 (1.01, 1.12)** |
| Region of Ontario  Greater Toronto Area (GTA) | Reference | Reference |
| Central East | **1.54 (1.45, 1.63)** | **1.62 (1.52, 1.72)** |
| Central West | **1.22 (1.18, 1.25)** | **1.25 (1.22, 1.29)** |
| Eastern | **1.18 (1.12, 1.24)** | **1.22 (1.16, 1.29)** |
| North East | 1.03 (0.99, 1.08) | **1.21 (1.15, 1.27)** |
| North West | **1.54 (1.34, 1.77)** | **1.45 (1.26, 1.67)** |
| South West | **1.74 (1.66, 1.82)** | **1.77 (1.69, 1.85)** |
| Preferred Language  English | Reference | Reference |
| Non-English | **0.84 (0.74, 0.96)** | 0.88 (0.77, 1.00) |
| Missing | **0.76 (0.73, 0.78)** | **0.79 (0.76, 0.82)** |

*Variables included in adjusted model: age group, material resources, COVID-19 wave, exposure setting, region of Ontario, and preferred language. Statistically significant estimates at p < 0.05 indicated in bold.

**Supplementary table 4: Unadjusted and adjusted odds of completing follow-up according to various characteristics, excluding participants returned to local PHUs due to wrong numbers, incomplete information, or ineligibility (N=126,935)**

| Comparison | Unadjusted estimates from separate bivariate analyses | Adjusted*  estimates from one multivariable analysis |
| --- | --- | --- |
| Age Group (years)  0 to 4 | **1.31 (1.19, 1.45)** | **1.26 (1.14, 1.40)** |
| 5 to 11 | **1.06 (1.00, 1.12)** | 1.03 (0.96, 1.10) |
| 12 to 19 | **1.06 (1.00, 1.12)** | 1.01 (0.94, 1.08) |
| 20 to 39 | Reference | Reference |
| 40 to 59 | **1.34 (1.25, 1.44)** | **1.31 (1.23, 1.41)** |
| 60 to 79 | **1.43 (1.30, 1.57)** | **1.41 (1.29, 1.56)** |
| 80+ | **1.23 (1.02, 1.49)** | **1.32 (1.09, 1.60)** |
| Material Resources  Quintile 1 | Reference | Reference |
| Quintile 2 | **0.92 (0.86, 0.98)** | **0.91 (0.85, 0.97)** |
| Quintile 3 | **0.80 (0.75, 0.86)** | **0.78 (0.73, 0.83)** |
| Quintile 4 | **0.73 (0.68, 0.78)** | **0.70 (0.66, 0.75)** |
| Quintile 5 | **0.61 (0.57, 0.65)** | **0.58 (0.55, 0.62)** |
| COVID-19 Wave  Wave 2 (01/09/2020-28/02/2021) | Reference | Reference |
| Wave 3 (01/03/2021-31/07/2021) | 0.97 (0.92, 1.02) | 0.97 (0.91, 1.02) |
| Wave 4 (01/08/2021-14/12/2021) | **1.10 (1.05, 1.16)** | **1.13 (1.07, 1.19)** |
| Wave 5 (15/12/2021-28/02/2022) | **0.57 (0.50, 0.64)** | **0.59 (0.52, 0.67)** |
| Exposure Setting  School | Reference | Reference |
| Commercial Establishment | 0.92 (0.83, 1.03) | **0.88 (0.78, 0.99)** |
| Congregate | **1.15 (1.10, 1.21)** | **1.11 (1.05, 1.18)** |
| Medical | 0.96 (0.85, 1.08) | **0.85 (0.75, 0.96)** |
| Other | **1.15 (1.09, 1.22)** | 1.05 (0.99, 1.12) |
| Workplace | 0.94 (0.86, 1.03) | **0.90 (0.81, 0.99)** |
| Region of Ontario  Greater Toronto Area (GTA) | Reference | Reference |
| Central East | **1.28 (1.16, 1.41)** | **1.33 (1.20, 1.47)** |
| Central West | 0.99 (0.94, 1.03) | **1.06 (1.01, 1.11)** |
| Eastern | 0.99 (0.92, 1.07) | **1.11 (1.02, 1.20)** |
| North East | **1.25 (1.14, 1.37)** | **1.34 (1.21, 1.47)** |
| North West | 1.20 (0.99, 1.46) | **1.36 (1.12, 1.65)** |
| South West | **1.64 (1.52, 1.77)** | **1.78 (1.64, 1.92)** |
| Preferred Language  English | Reference | Reference |
| Non-English | **0.79 (0.65, 0.96)** | 0.83 (0.68, 1.01) |

*Variables included in adjusted model: age group, material resources, COVID-19 wave, exposure setting, region of Ontario, and preferred language. Statistically significant estimates at p < 0.05 indicated in bold.

**Supplementary table 5: Unadjusted and adjusted odds of completing follow-up according to various characteristics, including participants with Disposition set to complete, but no Contact Tracing Complete task (N=144,851)**

| Comparison | Unadjusted estimates from separate bivariate analyses | Adjusted*  estimates from one multivariable analysis |
| --- | --- | --- |
| Age Group (years)  0 to 4 | **1.21 (1.13, 1.29)** | **1.12 (1.05, 1.20)** |
| 5 to 11 | **1.11 (1.07, 1.15)** | 0.97 (0.93, 1.01) |
| 12 to 19 | **1.07 (1.03, 1.11)** | 0.96 (0.92, 1.01) |
| 20 to 39 | Reference | Reference |
| 40 to 59 | **1.20 (1.15, 1.25)** | **1.18 (1.13, 1.23)** |
| 60 to 79 | **1.09 (1.03, 1.16)** | **1.12 (1.06, 1.19)** |
| 80+ | **0.84 (0.76, 0.93)** | 0.95 (0.85, 1.05) |
| Material Resources  Quintile 1 | Reference | Reference |
| Quintile 2 | **0.95 (0.91, 0.99)** | **0.94 (0.90, 0.98)** |
| Quintile 3 | **0.88 (0.85, 0.92)** | **0.87 (0.84, 0.91)** |
| Quintile 4 | **0.82 (0.78, 0.85)** | **0.81 (0.77, 0.84)** |
| Quintile 5 | **0.70 (0.67, 0.74)** | **0.68 (0.65, 0.71)** |
| COVID-19 Wave  Wave 2 (01/09/2020-28/02/2021) | Reference | Reference |
| Wave 3 (01/03/2021-31/07/2021) | **1.22 (1.18, 1.26)** | **1.25 (1.21, 1.29)** |
| Wave 4 (01/08/2021-14/12/2021) | **1.66 (1.61, 1.72)** | **1.69 (1.63, 1.75)** |
| Wave 5 (15/12/2021-28/02/2022) | 1.05 (0.95, 1.15) | 1.10 (1.00, 1.21) |
| Exposure Setting  School | Reference | Reference |
| Commercial Establishment | **0.88 (0.82, 0.94)** | **0.85 (0.79, 0.92)** |
| Congregate | **0.94 (0.91, 0.97)** | **0.96 (0.92, 1.00)** |
| Medical | **0.72 (0.67, 0.77)** | **0.69 (0.64, 0.75)** |
| Other | 1.00 (0.97, 1.04) | **0.92 (0.89, 0.96)** |
| Workplace | **0.89 (0.84, 0.95)** | **0.86 (0.80, 0.92)** |
| Region of Ontario  Greater Toronto Area (GTA) | Reference | Reference |
| Central East | **1.13 (1.06, 1.20)** | **1.12 (1.05, 1.19)** |
| Central West | **1.14 (1.10, 1.17)** | **1.12 (1.08, 1.15)** |
| Eastern | **0.93 (0.89, 0.98)** | **0.86 (0.82, 0.91)** |
| North East | **1.28 (1.21, 1.36)** | **1.20 (1.13, 1.28)** |
| North West | 0.99 (0.88, 1.11) | 1.10 (0.98, 1.24) |
| South West | **1.22 (1.17, 1.28)** | **1.27 (1.21, 1.32)** |
| Preferred Language  English | Reference | Reference |
| Non-English | **0.68 (0.60, 0.77)** | **0.76 (0.67, 0.85)** |

*Variables included in adjusted model: age group, material resources, COVID-19 wave, exposure setting, region of Ontario, and preferred language. Statistically significant estimates at p < 0.05 indicated in bold.

# References

1. Chambers A, Quirk J, MacIntyre EA, Bodkin A, Hanson H. Lessons learned from implementing a surge capacity support program for COVID-19 contact management in Ontario. Can J Public Health. 2023;114(4):555-62.

2. Ontario Government. Ontario updating public health measures and guidance in response to Omicron. Queen's Printer of Ontario. December 30, 2021. https://news.ontario.ca/en/release/1001386/ontario-updating-public-health-measures-and-guidance-in-response-to-omicron, Accessed 16 Jan 2025.
